# The Crippen Case

**Published:** 1910-10-29

**Authors:** 


### The Hospital

A Journal of

The Medical Sciences and Hospital Administration.

New Series. No. 194, Vol. VIII. [No. 1266, Vol. XLIX.] SATURDAY, OCTOBER 29, 1910.

### THE CRIPPEN CASE.

In the remarkable trial which ended last Saturday in a verdict of guilty and a sentence of death there arose much of unusual medical and medico-legal interest and importance. The dramatic events of Crippen's flight with his paramour, his detection on the *Montrose*, the use of wireless telegraphy, and the pursuit by Inspector Dew are merely incidentals from the medical standpoint, though naturally they enhance the human interest in the case which members of the profession, like everybody else, are bound to take.

First and foremost the prisoner describes himself, and is commonly spoken of, as "Dr." Crippen. It is fairly common knowledge that this title is one conferred merely by the "Cleveland Homœopathic Hospital" (U.S.A.), whatever that may import; but undoubtedly a section of the public which reads its papers carelessly will go about with the impression that he is a qualified medical man in the genuine sense. It came out during the prisoner's cross-examination that in obtaining this degree of M.D. he received no training in practical surgery. It is true that no reliance can be placed upon anything that he said, and that he had a definite motive for disclaiming any knowledge of practical surgery; but the point is that the scandals connected with the acquisition of medical degrees in America are so many and so grave that it is impossible to distinguish the true from the Lamson-Crippen brands. It is, further, to be remembered that Crippen pursued in London the callings of agent and manager for an American patent-medicine vending concern, owner of a dentistry business, and eye throat and nose specialist. He alleges that he dealt with two hundred letters a day, and admits that he undertook the care of hundreds of patients whom he had never seen. The thousands of this criminal's victims who have paid for and swallowed his "remedies" may possibly now repent their folly and eschew it for the future. But the gullibility of mankind is so enormous that it is quite on the cards that the notoriety which the quack firm has received may actually swell its receipts.

There are several questions of medical significance upon which, perhaps inevitably, the defence indulged in a darkening of counsel. One is the amount of anatomical skill necessary to remove the thoracic viscera in one piece. Every country practitioner knows quite well that this is a feat which anyone who has seen it done a few times can perform, and that no special training in post-mortem work other than the most casual attendance in the dead-house during student days has enabled him to tackle this unpleasant job when suddenly faced with the necessity for it in after years. In the matter of the much-talked-of scar, it seems as if the operation wound had either suppurated or the scar stretched in course of years; for it is described as a wide band, broader below than above. The comments of the Lord Chief Justice upon the unsatisfactory discrepancy between the evidence about this scar of the medical experts for the defence, as given before the magistrate and before himself respectively, seem to be justified. It is a very great pity when men of some distinction in the profession thus invite a snubbing from so fair and wise a judge.

Then there are the points connected with hyoscine. The defence were inclined to make capital out of the admitted fact that the Home Office experts were made cognisant of Crippen's purchase of this drug a fortnight before the disappearance of Belle Elmore. Unless it be assumed that Dr. Willcox is either incompetent or dishonest, it seems eminently right that every possible help should thus be given him when analysing human remains for suspected poison. The fatal doses of some poisons are so small that their detection is a matter of the utmost difficulty, and in the interests of justice it is essential that every hint or clue should be taken advantage of. Then, again, questions were put regarding two tests which the analyst had not applied. For the melting-point test the entire quantity of hyoscine recovered was not nearly enough, and for the other test it would just have sufficed; in the latter case the predicament of the analyst may be imagined who should get a negative result from the test for one single poison and exhaust his whole available material in doing it. If the melting-point test could be insisted on, murderers might almost rely on going scot-free, provided they refrained from their crimes in public. Much was heard of the infrequency with which hyoscine is used in England, and Crippen even ventured to pretend that this is a valuable drug whose virtues are known almost solely to Americans. The plain fact is that it has been used largely in asylums, and that under another name* (scopolamine) it is widely used in the United Kingdom for combination with morphine preliminary to general anæsthesia and during labour. As for the twelve thousand homœopathic doses of it which the prisoner alleges he dispensed between January and July, it is sufficient that he produced not a single individual who had swallowed one. The public will do well to remember that the other alkaloid contained in henbane, hyoscyamine to wit, is widely advertised and sold as a preventive of sea-sickness. If they will also realise that this drug is almost as dangerous as hyoscine itself, and that as prepared and sold for sea-sickness it probably contains both, they may be inclined to avoid henceforth a particularly dangerous and harmful preparation which is much too widely used and has many potentialities of evil. If they do this, it may be that the sordid history and tragic end of Cora Crippen may yet achieve some valuable purpose.

* Hale White, *Materia Medica*, 11th Edition, p. 370, where the alkaloid as well as its hydrobromide is described as a white crystalline powder, not a gummy substance.

